# A vicious circle between insulin resistance and inflammation in nonalcoholic fatty liver disease

**DOI:** 10.1186/s12944-017-0572-9

**Published:** 2017-10-16

**Authors:** Zhonge Chen, Rong Yu, Ying Xiong, Fangteng Du, Shuishan Zhu

**Affiliations:** 1 Medical Center of The Graduate School, Nanchang University, Nanchang, China; 2 Department of Endocrinology, Second Affliated Hospital, Nanchang University, Nanchang, China; 3 Department of Gastroenterology, Second Affliated Hospital, Nanchang University, No. 1, Minde Road, Nanchang, 330006 China

## Abstract

Nonalcoholic fatty liver disease (NAFLD) comprises a spectrum of diseases, including simple steatosis, nonalcoholic steatohepatitis (NASH), liver cirrhosis and hepatocellular carcinoma. Lipotoxicity, insulin resistance (IR) and inflammation are involved in the disease process. Lipotoxicity promotes inflammation and IR, which in turn, increase adipocyte lipolysis and exacerbates lipotoxicity. Furthermore, IR and inflammation form a vicious circle, with each condition promoting the other and accelerating the development of NAFLD in the presence of lipotoxicity. As an integrator of inflammatory pathway networks, nuclear factor-kappa B (NF-κB) regulates expression of pro-inflammatory cytokines, such as tumor necrosis factor-alpha (TNF-α), interleukin 6 (IL-6), and anti-inflammatory cytokines, such as adiponectin in NAFLD. In this review, the relationships between lipotoxicity, IR and inflammation in NAFLD are discussed, with particular emphasis on the inflammatory pathways.

## Background

**Fig. 1:**
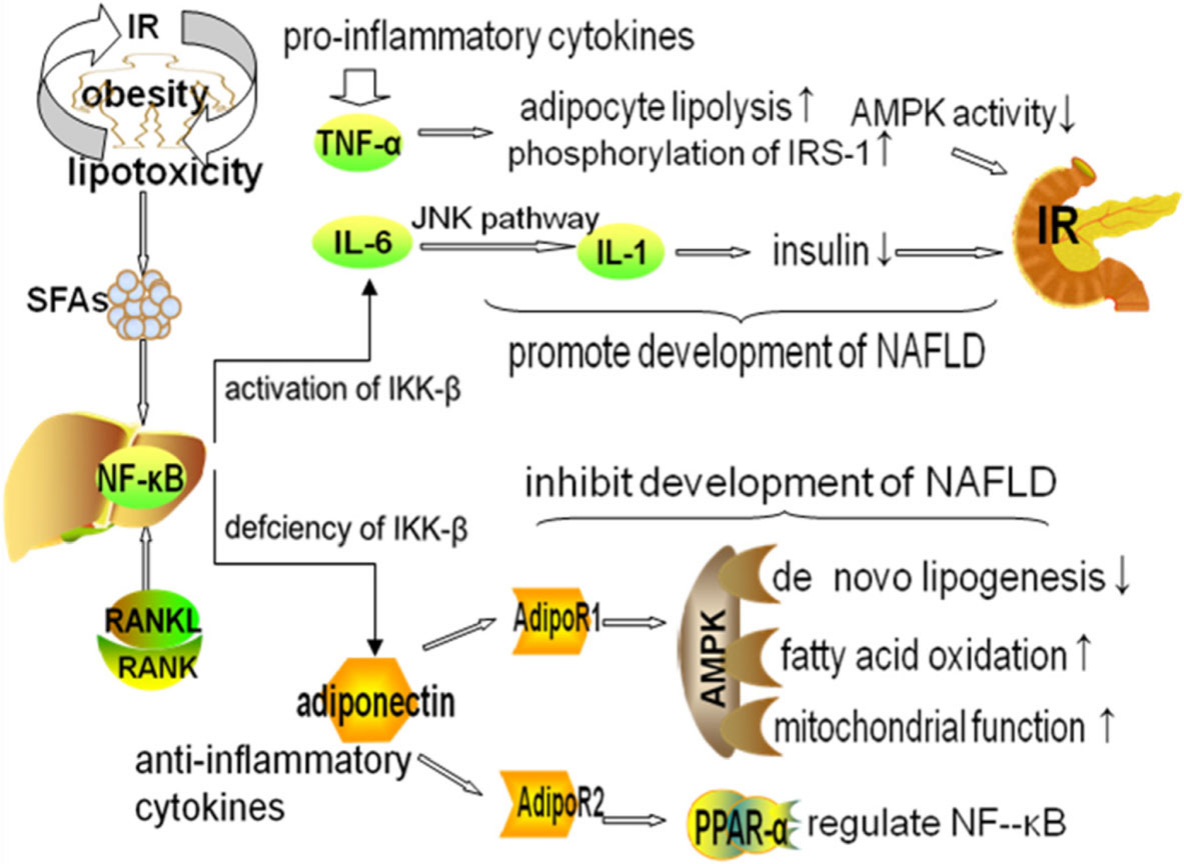
NAFLD related lipotoxicity, IR and inflammation. Legend 1: Lipotoxicity promotes inflammation and insulin resistance (IR). In turn, IR increases adipocyte lipolysis and exacerbates lipotoxicity. By binding with specific receptors, saturated fatty acids (SFAs) activate nuclear factor-kappa B (NF-κB). In IR, liver expression of NF-κB is extremely high. Receptor activator of NF-κB (RANKL) binds to its receptor (RANK) in liver and activates the NF-κB pathway. Activation of NF-κB kinase-β (IKK-β) promotes expression of pro-inflammatory cytokines, such as tumor necrosis factor-alpha (TNF-α) and interleukin 6 (IL-6). TNF-α increases adipocyte lipolysis, strengthens phosphorylation of insulin receptor substrate-1(IRS-1) and reduces AMPK activity. IL-6 activates the c-Jun N-terminal kinase (JNK) pathway and suppresses IL-1 induced secretion of insulin. TNF-α and IL-6 promote development of IR and NAFLD. Defciency of IKK-β promotes expression of anti-inflammatory cytokines, such as adiponectin. Adiponectin receptor 1 (AdipoR1) activates AMPK activity, which then suppresses DNL, increases fatty acid oxidation and promotes mitochondrial function. AdipoR2 activates peroxisome proliferator-activated receptor-alpha (PPAR-α) signaling, which exerts anti-inflammatory effects by regulating NF-κB. Adiponectin inhibits the development of IR and NAFLD

Nonalcoholic fatty liver disease (NAFLD) is one of the most common liver diseases worldwide. It covers a spectrum of diseases, including simple steatosis, nonalcoholic steatohepatitis (NASH), liver cirrhosis and hepatocellular carcinoma [1]. NASH refers to the presence of hepatic steatosis and inflammation with hepatocyte injury (ballooning) in the presence or absence of fibrosis. In humans, NAFLD is a necessary precursor of metabolic syndrome, rather than being a mere “manifestation of the metabolic syndrome” [2]. NAFLD is a significant health issue, because it not only affects up to 30% of adults and up to 10% of children in developed countries [3], but is also predicted to become the leading indication for liver transplantation in the future [4]. Current studies focus on elucidating the factors that drive the progression from simple steatosis to NAFLD. The pathogenesis of NAFLD was originally described by the “two-hit theory” in which the first hit is represented by an accumulation of fatty acids and triglycerides in liver. The second hit is represented by chronic stresses, such as enhanced lipid peroxidation, generation of reactive oxygen species (ROS), endoplasmic reticulum stress (ERS), and byproducts of exacerbated pro-inflammatory responses in fatty liver [5]. IR is recognized as a critical pathophysiological factor in NAFLD. Nevertheless, the mechanisms underlying NAFLD remain to be fully elucidated. IR, lipotoxicity and inflammation are all known to be involved in the disease process [6]. However, “vicious circle” represented by the mutual positive feedback regulation that exists between IR and inflammation cannot be ignored since these responses act in combination to promote the development of NAFLD in the presence of lipotoxicity. This review will highlight the relationships among lipotoxicity, IR and inflammation in NAFLD, as illustrated in Fig. 1. Further understanding of the associations among these responses will provide a basis for the identification of novel therapeutic targets for NAFLD.

## Lipotoxicity

Adipose tissue is physiologic reservoir of fatty acids [2]. When storage ability is overwhelmed, the endocrine functions of adipose tissues are altered and the ensuing accumulation of ectopic fat leads to lipotoxicity, which promotes low-grade inflammation and IR in the liver [7]. At present, lipotoxicity is regarded as the driving force in the mechanism underlying disease progression from simple steatosis to NASH [8]. Fatty liver can be generated by mechanisms including: increased free fatty acids (FFAs); increased intake of dietary fat; increased de novo lipogenesis (DNL); decreased free fatty oxidation and; decreased hepatic triglycerides secretion [9].

## Free fatty acids

Lipotoxic injury appears to occur because of excessive levels of FFAs in hepatocytes [8]. Circulating FFAs, which are the primary source of hepatic fat accumulation in NAFLD, are primarily derived from adipose tissue lipolysis and partly from excess lipoproteins. In the fasting state, FFAs represent a major fuel substrate for all tissues except the brain in the fasting state [10]. Plasma concentrations of FFAs are high during fasting, but decline after feeding due to the anti-lipolytic action of insulin. Under IR conditions, high FFA levels are caused by resistance to the anti-lipolytic action of insulin [11]. IR plays a key role in lipolysis in adipose tissue, causing trafficking of superfluous FFAs and promoting the development of lipotoxicity. In humans, a short-term rise in FFAs leads to hepatic IR [12]. Furthermore, FFAs interact with insulin signaling, thereby contributing to the IR [13]. The anti-lipolytic function of insulin is impaired in the context of IR, which may facilitate hepatic triglyceride synthesis. FFAs deposited in the liver and heart are known as ectopic fat [14]. Deposition of hepatic lipids promotes the development of NAFLD.

## Saturated fatty acids

Under physiological conditions, saturated fatty acids (SFAs) are stored as lipid droplets, transferred into mitochondria for β-oxidation, and secreted into blood plasma as very low-density-lipoproteins [15]. The superfluous SFAs generate lipotoxic intermediate products, such as diacylglycerols [8]. Intrahepatic diacylglycerol content is negatively associated with hepatic insulin sensitivity in patients with NAFLD complicated by obesity [5]. Lipotoxic intermediate products cause ERS, accumulation of unfolded or misfolded proteins and formation of ROS, all of which result in apoptosis, a major factor in the pathogenesis of NASH [15]. SFAs induce an ERS response in hepatocytes and increase ERS in patients with NAFLD [16]. By binding toToll-like receptor 4, SFAs stimulate a suite of cascaded reactions that result in effects, such as augmentation of mitochondrial dysfunction and activation of pro-inflammatory nuclear factor-kappa B (NF-κB) [15].

## Triglycerides

Plasma FFAs are reabsorbed in various organs where, if not oxidized, they accumulate in the form of triglycerides and promote cell lipotoxicity and mitochondrial dysfunction [10]. Triglycerides are a major form of lipids stored in the liver of NAFLD patients. Although epidemiological studies suggest triglyceride-mediated pathways have negative influences on disease [17], recent evidence indicates that trigylcerides have protective activity. Diacylglycerol acyltransferase 1 and 2 (DGAT1/2) catalyze the final step in triglyceride synthesis. Obese mice overexpressing DGAT1 in adipocytes and macrophages are protected from activation and accumulation of macrophages, systemic inflammation and IR [18]. Inhibition of triglyceride synthesis via DGAT2 antisense oligonucleotides leads to an amelioration of hepatic steatosis, but aggravates hepatic cell damage [19]. Triglycerides synthesis seems to be an adaptive, protective response in hepatocytes. Therefore, triglycerides accumulation in the liver cannot be considered as a pathologic response, but rather as a physiologic response to increased caloric consumption.

## Insulin resistance

Under normal conditions, the β-cells of the pancreas secrete insulin after a meal or after the release of hormone, such as catecholamines and glucagon, along with change in plasma glucose concentrations [11]. Insulin mediates precise regulation of glucose metabolism and plasma concentrations, not only by promoting glucose uptake by skeletal muscle, liver and adipose tissue, but also by suppressing hepatic glucose production. Insulin plays an important role in lipid metabolism by combining with its receptor to promote fatty acid esterification, fatty acid storage in lipid droplets and also inhibit lipolysis. Insulin also increases DNL [20] leading to enhanced palmitate synthesis in NAFLD patients, which increases the risk of lipotoxicity andcell damage.

IR increases adipocyte lipolysis and circulating FFAs and reduces hepatic glycogen storage, which promotes gluconeogenesis in NAFLD patients. Hyperinsulinemia may be a response to systemic IR, which augments hepatic DNL [21]. Intrahepatic lipid accumulation is increased and triglycerides are secreted in the form of very-low-density lipoproteins. The accumulating lipids are transported to adipose tissue, reducing the ability of adipocytes to store lipids. Lipotoxicity impairs insulin signaling, induces oxidative damage, and promotes inflammation and fibrosis [22], which is thought to be associated with the progression from simple steatosis to NASH, liver fibrosis and hepatocellular carcinoma in NAFLD patients.

Under conditions of IR, abnormally high insulin levels are required to metabolize glucose and inhibit hepatic glucose production effectively due to the reduced insulin sensitivity of the peripheral tissues. In the context of IR, the pancreas is stimulated to increase insulin secretion into the portal vein, leading to higher insulin levels in the liver than in the periphery. High concentrations of hepatic glucose and plasma insulin are recognized as biomarkers of hepatic IR [23]. Elevated fasting glucose results from hepatic IR, whereas increased FFAs concentrations are caused by peripheral IR [24]. Some NAFLD patients have normal fasting glucose concentrations, but high fasting insulin concentrations and hepatic IR. IR is recognized as the critical pathophysiological factor in NAFLD. Hepatic IR contributes to steatosis of NAFLD by impairing insulin receptor substrate1/2 (IRS-1/2) tyrosine phosphorylation [25]. FFAs interact with insulin signaling, thereby contributing to IR.

## Inflammation

In addition to the influence of abnormalities in lipid metabolism, inflammation also contributes to IR. Pro-inflammatory cytokines and transcription factors are highly expressed in adipose tissue and liver. Obesity, which is a state of chronic low-grade inflammation and a risk factor for IR and NAFLD, is induced by over-nutrition and is a primary cause of decreased insulin sensitivity. Obesity leads to lipid accumulation and activates the c-Jun N-terminal kinase (JNK) and nuclear factor-kappa B (NF-κB) signaling pathways, which consequently increase production of pro-inflammatory cytokines, such as tumor necrosis factor-alpha (TNF-α) and interleukin-6 (IL-6) [26]. In addition, various adipose tissue-derived proteins, such as adiponectin and leptin, are considered to be major links between obesity, IR and related inflammatory disorders.

## Nuclear factor-kappa B

NF-κB is a transcription factor that is involved in innate and adaptive immune responses as well as a series of pathological processes, such as inflammation [27]. Under normal conditions, NF-κB is sequestered in the cytoplasm and binds to IκB proteins, which then inhibits nuclear localization of NF-κB. Activation of NF-κB is normally moderate, whereas, under conditions of IR, its expression in liver and adipose tissue is hugely increased [28]. The inhibitor of NF-κB kinase (IKK) complex plays an important role in activation of NF-κB by phosphorylating inhibitory molecules. The IKK complex, comprising IKKα and IKKβ, is activated in response to stimulation by pathogenic stimuli. This induces phosphorylation and degradation of the NF-κB inhibitor α (IκBα), then exposing the nuclear localization sequence of NF-κB. As a consequence, NF-κB is translocated to the nucleus leading to upregulation of the expression of target genes encoding inflammatory mediators, such as TNF-α and IL-6 [27].

Several signaling pathways, such as the IKKβ/NF-κB pathway, are involved in the pathogenesis of IR [29].The IKK-β pathway has been demonstrated to be a target for TNF-α-induced IR in mice and in cell lines [30]. Chronic hepatic inflammation in a hepatic IKK-β transgenic mouse model resulted in low level activation of NF-κB and modest systemic IR [30]. Liver-specific IKK-β knockout mice fed a high-fat diet retained liver insulin function [31]. On the one hand, IKK-β deficiency in adipocytes inhibits FFA-induced expression of TNF-α and IL-6, while the other hand, IKK-β activation prevents expression of anti-inflammatory cytokines, such as adiponectin [32]. Eelevated NF-κB activity in hepatic cells is associated with IR. Deletion of IKK-β ameliorates glucose tolerance and insulin sensitivity. Thus, treatments inhibiting the NF-κB pathway may alleviate IR.

Receptor activator of NF-κB (RANKL) regulates hepatic insulin sensitivity [33]. Blockade of RANKL signaling in hepatocytes improves insulin sensitivity and normalizes glucose concentrations. Soluble RANKL is produced by many tissues including skeletal muscle, several immune cell types and adipose tissue. RANKL binds to its specific receptor (RANK) in liver and activates the NF-κB pathway, which then increases local inflammation and leads to IR [34]. It can be speculated that RANKL might target the liver as a key organ of metabolism, thereby contributing to hepatic IR.

## Tumor necrosis factor-alpha

TNF-α is an adipose tissue-derived pro-inflammatory cytokine. Increased TNF-α production is a consequence of metabolic disturbances and TNF-α expression is high in obese animals. Relationships between TNF-α and IR are formed by increasing both adipocyte lipolysis and serine/threonine phosphorylation of IRS-1 [35]. IR is enhanced by antibody-mediated neutralization of TNF-α [36]. Insulin sensitivity is increased in mice lacking TNF-α. Because TNF-α can increase glucose uptake in both visceral and subcutaneous adipocytes, modulating TNF-α signaling may be a therapeutic approach for IR [37]. TNF-α expression in NASH patients is higher than that in patients with simple steatosis. More advanced fibrosis is accompanied by increased TNF-α expression [38]. In addition, TNF-α reduces AMP-activated protein kinase (AMPK) activity [39], which may contribute to the development of NAFLD.

## Interleukin-6

IL-6 is secreted mainly by adipose tissue and is recognized as an inflammatory mediator. Treatment of obese mice with anti-IL-6 antibodies leads to increased insulin sensitivity indicating that this cytokine is involved in the pathogenesis of hepatic IR [40]. IL-6 inhibits insulin-mediated lipolysis in white adipose tissue and increases the delivery of FFAs to liver. Compared to lean individuals, obese adolescents with IR have higher adipose tissue IL-6 concentrations than lean individuals [41]. Furthermore, IL-6 activates the NF-κΒ-JNK-ceramide pathway, which in turn inhibits insulin signaling and increases gluconeogenic protein transcription. JNK exists as JNK-1, −2, and −3 isoforms, which modulate pro-inflammatory cytokine production, karyomitosis, and cellular apoptosis, thus representing associations with inflammation and IR [42]. Suppression of JNK ameliorates IR and glucose tolerance. JNK plays a significant role in IR by suppressing secretion of insulin from pancreatic β-cells via pro-inflammatory stimuli, such as IL-1. Excessive activation of JNK in peripheral insulin-sensitive tissues accelerates IR [43]. JNK-1 deficiency in adipose tissue protects against hepatic steatosis and improves glucose intolerance, insulin clearance and IR. Inhibition of JNK decreases the release of IR-related pro-inflammatory cytokines, such as TNF-α [44]. Overall, further researches are required to clarify the relationship between JNK and IR.

## Adiponectin

Adiponectin is produced primarily by white adipose tissue and is detected in the circulation in various isoforms, such as full-length (low, medium and high molecular weight isoforms) and globular fragments. This adipokine acts as an anti-inflammatory cytokine in obesity and IR, which are associated with decreased levels, but as a pro-inflammatory cytokine in osteoarthritis and type 1 diabetes mellitus, which are associated with increased levels [45]. Weight loss induces adiponectin synthesis [46]. Expression of hepatic adiponectin is decreased in NASH patients while expression of hepatic adiponectin and its receptors are increased after weight loss [47]. Chronic overexpression of adiponectin results in increased subcutaneous fat and protects against diet-induced IR [48]. Decreased expression of adiponectin receptors is detected in IR in vivo, indicating that adiponectin activity is impaired by the expression of its cognate receptor [49]. The insulin-sensitizing activity of adiponectin is mediated by upregulating peroxisome proliferator activated receptor-alpha (PPAR-α) and its target genes, including CD36, ACO, and UCP-2, in liver [50]. Activation of PPAR-α in mice model of obese diabetes using a specific agonist stimulates adiponectin potency and adiponectin receptor expression, thus rescuing these mice from obesity-induced IR [51].

Adiponectin has two receptors associated with glucose metabolism, which connects adiponectin with the amelioration of IR. Adiponectin receptor 1 (AdipoR1) decreases the expression of genes encoding hepatic gluconeogenic enzymes and molecules involved in lipogenesis by activating AMPK. Adiponectin receptor 2 (AdipoR2) upregulates the expression of genes associated with glucose consumption by activating PPAR-α signaling [52]. The glucose-lowering effect of adiponectin is mediated by suppressing gluconeogenesis or glycogenolysis. In mice model, short-term infusion of adiponectin resulted in suppression of endogenous glucose production by suppressing glucose-6-phosphatase mRNA and phosphoenol pyruvate carboxykinase mRNA in liver [53]. Overexpression of adiponectin protects against high-fat diet-induced lipotoxicity and increases the metabolic flexibility of adipose tissue in mice [54]. Adiponectin ameliorates hepatic IR by reducing glycogenesis and lipogenesis and increasing glucose consumption.

Adiponectin knockout mice show high TNF-α mRNA expression in adipose tissue and high TNF-α protein concentrations in the circulation, indicating that adiponectin exerts anti-inflammatory activity [55], which is mediated not only by suppression of TNF-α expression, but also induction of anti-inflammatory gene expression in human leukocytes, including IL-10 and IL-1 receptor antagonist [56]. TNF-α inhibits the transcription of adiponectin in adipocytes, thereby negatively influencing inflammation. In addition, adiponectin can ameliorate alcohol- and obesity-associated liver abnormalities, such as hepatomegaly and steatosis, by enhancing the activity of carnitine palmitoyltransferase I and oxidation of hepatic fatty acid, while decreasing the activity of acetyl-CoA carboxylase and fatty acid synthase, two key enzymes involved in fatty acid synthesis [57].

## Leptin

Leptin, which is derived predominantly from white adipose tissue, inhibits appetite, increases fatty acid oxidation, and decreases glucose, body fat and weight. Leptin levels are influenced by nutrition and its signal is transmitted by the Janus kinase signal transducer and activator of transcription (JAK-STAT) pathway [58]. Leptin resistance, defined by reduced ability of leptin to suppress appetite and weight gain, is often observed in obese individuals and serum levels of leptin decrease with reductions in body weight. Leptin resistance can be overcome by certain adipose tissue-derived factors, such as fibroblast growth factor 1. Administration of fibroblast growth factor 1 in NAFLD mice ameliorates hepatic steatosis. This factor can not only act as a potent glucose-lowering and insulin-sensitizing agent but also regulate hepatic lipid metabolism [59].

Leptin-associated appetite and energy homeostasis are associated with progression of IR [60], indicating that leptin plays a role in exacerbating IR. The association of serum leptin concentrations with NAFLD in pre-diabetic subjects is regulated by insulin secretory dysfunction and IR [61]. Although metformin is not proven to be a valid therapy in human NASH, it is able to upregulate leptin receptor expression in mice [62]. Although increased soluble leptin receptor levels are also detected in patients with type 2 diabetes after metformin treatment, the relationship between leptin and IR requires further investigation.

The role of leptin in regulating inflammation has become evident over recent years [63]. Leptin exerts pro-inflammatory activity in models of auto-inflammatory or immune-mediated inflammatory disorders. Leptin induces expression of inflammatory cytokines, which in turn, stimulates the release of leptin from adipocytes. Increased serum leptin concentrations are associated with severity of liver diseases, such as inflammation and fibrosis [64]. Increased serum leptin concentrations were detected in a prospective NAFLD study [65]. A recent meta-analysis of 33 studies with 2612 individuals summarized the current evidence for the role of leptin in NAFLD [66]. This analysis revealed higher serum leptin concentrations in patients with simple steatosis compared with controls and showed a correlation between higher leptin concentrations and increased severity of NAFLD.

The adiponectin/leptin ratio is implicated as a biomarker of adipose tissue dysfunction and correlates with IR more closely than either adiponectin or leptin alone. In a clinical study with sample of 140 Caucasian subjects, the adiponectin/leptin ratio was dramatically decreased in metabolic syndrome, while markers of inflammation and oxidative stress increased in these subjects [67].This study concluded that the adipose tissue dysfunction indicated by low adiponectin/leptin ratios might promote oxidative stress and inflammation.

## Peroxisome proliferator-activated receptors

As integrators of inflammatory and metabolic pathway networks, PPARs are lipid sensors that regulate metabolic processes [68]. There are three PPAR isotypes, PPAR-α, PPAR-β/δ and PPAR-γ with different tissue distribution patterns and ligand specificities. PPAR-α is highly expressed in liver, kidney, and muscle, while PPAR-γ isexpressed mainly in adipose tissue and PPAR-β/δ is expressed ubiquitously [69]. PPAR-α, which is important in regulation of fatty acid uptake, β-oxidation, ketogenesis, bile acid synthesis, and triglyceride turnover [70] is activated by fibrates that have therapeutic function for hypertriglyceridemia. In addition to its function in the regulation of metabolism, PPAR-α exerts anti-inflammatory effects by regulating NF-κB [71]. A high-fat diet is related to high liver expression of PPAR-α, which is involved in fatty acid oxidation, and represents a protective response. A clinical study showed that PPAR-α gene expression in human liver is negatively associated with NASH severity [72]. Lifestyle interventions and bariatric surgery achieve amelioration of liver histology along with an increased expression of PPAR-α and its target genes. In the context of a high-fat diet, PPAR-α knockout mice have a significantly higher NAFLD activity score [73]. In a mouse model of NASH, treatment with a PPAR-α agonist (Wy-14,643) reverses fibrosis and NASH [74]. Activation of poly (ADP-ribose) polymerase 1 (PARP1) in fatty liver prevents activation of fatty acid oxidation by inhibiting PPAR-α signaling. Thus, pharmacological inhibition of PARP1 may alleviate suppression of PPAR-α and therefore, have potentially therapeutic effects in NAFLD.

## AMP-activated protein kinase

AMPK, which is a heterotrimeric complex of an -α, −β and -γ subunit, is a member of serine/threonine kinase family and was initially isolated from liver. However, all three subunits are expressed in various organs, including heart, lung, brain, and kidney [75]. The liver primarily expresses α1, α2, γ1, and γ2 subunits. From the N-terminus to the C-terminus, the α-subunit is composed of a kinase domain, an auto-inhibitor domain and α-subunit carboxy-terminal domain. Adenosine monophosphate or adenosine diphosphate binding promotes phosphorylation of AMPK and increases its activity. The auto-inhibitor domain of the α-subunit decreases AMPK activity in the absence of adenosine monophosphate. Phosphorylation of the ST loop, which consists of a serine/threonine-rich insert of amino acids in the α-subunit, may lower AMPK activity [76]. In mouse models, AMPK overexpression facilitates expression of small heterodimer partner mRNA in primary hepatocytes and ameliorates hepatic IR [77]. Compared to healthy individuals, AMPK activity is lower in patients with advanced fibrosis/cirrhosis [78].

AMPK is required to maintain mitochondrial function in adipose tissue and protects against obesity-induced NAFLD [79]. Hepatic AMPK is also significant in preventing liver lipid accumulation and IR. A clinical study revealed that AMPK activity is lower in adipose tissue of obese patients with IR than in BMI-matched insulin-sensitive individuals, indicating that adipose tissue AMPK is important in NAFLD [80]. The mechanism by which AMPK activity is decreased in adipose tissue in obese IR patients remains to be clarified. It can be speculated that this effect is mediated by decreased circulating levels of adiponectin [81] and altered lipolysis [82] because increases in both are shown to activate AMPK. Another possibility is that inflammatory factors known to be elevated in NAFLD, such as TNF-α, reduce AMPK activity [83]. AMPK activation exacerbates NAFLD by suppressing DNL and increasing fatty acid oxidation in liver, and promoting mitochondrial function in adipose tissue.

DNL is involved in the metabolic pathway that is responsible for transformation of carbohydrate to fatty acids. For DNL, ATP citrate lyase generates acetyl-CoA that is then converted to malonyl-CoA via acetyl-CoA carboxylase (ACC). DNL contributes 5% to liver triglyceride content in healthy individuals, but contributes 26% in individuals with NAFLD [84], an increase of approximately five-fold that indicates a relationship between the DNL pathway and NAFLD. AMPK phosphorylation of ACC blocks its dimerization, which then causes a reduction in ACC activity, inhibition of DNL and an increase in mitochondrial fatty acid oxidation [85].

Plasma FFA levels are increased in NAFLD patients and contribute to the increased liver lipid content. AMPK activation increases fatty acid oxidation by promoting carnitine palmitoyltransferase I flux [86] and NAFLD is ameliorated by increased liver fatty acid oxidation [87]. Treatment with small molecules that bind to ACC and imitate the inhibitory effects of AMPK phosphorylation on ACC activity inhibits DNL, increases fatty acid oxidation and alleviates NAFLD and IR [88]. The reduction of adipose tissue AMPK decreases mitophagy, which is an evolutionarily conserved quality control pathway that induces engulfment of damaged mitochondria into the autophagosome and degradation via fusion with a lysosome, leading to impaired adipose tissue mitochondrial function [89]. Mitochondrial dysfunction suppresses fatty acid oxidation in brown adipose tissue, causing redirection of fatty acids toward peripheral tissues, such as liver [90]. Therefore, maintenance of mitochondrial function in adipose tissue protects against the progression of IR and NAFLD. Thus, strategies to increase adipose tissue AMPK and improve mitochondrial function may alleviate the development of NAFLD.

## Conclusion

Recent advances in our understating of the physiopathology of NAFLD have revealed the complex mechanisms of this disease. Although the involvement of lipotoxicity, IR and inflammation in development of NAFLD is well-stablished, the associations among these remain to be elucidated. Here, we summarize the evidence that: 1) lipotoxicity promotes inflammation and IR; 2) IR aggravates lipotoxicity; 3) IR and inflammation are subject to mutual positive regulation. Elucidation of the “bridge” between IR and inflammation, as well as strategies to break this “bridge” will be important in developing novel treatments for NAFLD. Moreover, although the imbalance between pro-inflammatory and anti-inflammatory cytokines in NAFLD is well-described, a comprehensive analysis of the imbalance and strategies to reinstate the balance may offer opportunity for therapy of NAFLD.

## Abbreviations

ACC: acetyl-CoA carboxylase
AdipoR1/2: adiponectin receptor 1/2
AMPK: AMP-activated protein kinase
DGAT1/2: Diacylglycerol acyltransferase 1/2
DNL: de novo lipogenesis
ERS: endoplasmic reticulum stress
IKK: inhibitor of NF-κB kinase
IL-6: interleukin 6
IR: insulin resistance
IRS-1/2: insulin-stimulated insulin receptor substrate1/2
IκBα: NF-κB inhibitor α
JAK-STAT: Janus kinase, signal transducer and activator of transcription
NAFLD: nonalcoholic fatty liver disease
NASH: nonalcoholic steatohepatitis
NF-κB: nuclear factor-kappa B
PARP1: poly (ADP-ribose) polymerase 1
PPAR-α: peroxisome proliferator activated receptor-alpha
RANK: receptor of RANKL
RANKL: receptor activator of NF-κB
ROS: reactive oxygen species
SFAs: saturated fatty acids
TNF-α: tumor necrosis factor-alpha
