# Correction to: A vicious circle between insulin resistance and inflammation in nonalcoholic fatty liver disease

**DOI:** 10.1186/s12944-018-0678-8

**Published:** 2018-02-23

**Authors:** Zhonge Chen, Rong Yu, Ying Xiong, Fangteng Du, Shuishan Zhu

**Affiliations:** 1 Department of Gastroenterology, Second Affiliated Hospital, Nanchang University Nanchang, Nanchang, China; 2 Department of Endocrinology, Second Affliated Hospital, Nanchang University, Nanchang, China; 3 Department of Gastroenterology, Second Affliated Hospital, Nanchang University, No. 1, Minde Road, Nanchang, 330006 China

## Correction

Following publication of the original article [1], the corresponding author reported that he had mistyped the first author's unit. The affiliation of Zhonge Chen, “Medical Center of the Graduate School, Nanchang University, China”, should be changed to “Department of Gastroenterology, Second Affiliated Hospital, Nanchang University Nanchang, China”. All the other authors have agreed to this change. The corrected version should be as follows:

Chen Z^1^*, Yu R^2^*, Xiong Y^3^*, Du F^4^, Zhu S^5^.

^1^Department of Gastroenterology, Second Affliated Hospital, Nanchang University, Nanchang, China.

^2^Department of Endocrinology, Second Affliated Hospital, Nanchang University, Nanchang, China.

^3^Department of Gastroenterology, Second Affliated Hospital, Nanchang University, No. 1, Minde Road, Nanchang, 330006, China.

^4^Department of Gastroenterology, Second Affliated Hospital, Nanchang University, No. 1, Minde Road, Nanchang, 330006, China. 296978043@qq.com.

^5^Department of Gastroenterology, Second Affliated Hospital, Nanchang University, No. 1, Minde Road, Nanchang, 330006, China. 478675878@qq.com.

*Equal contributors.

Address correspondence to

ShuiShan Zhu, Department of Gastroenterology, Second Affliated Hospital, Nanchang University, No. 1, Minde Road, Nanchang 330006, China. E-mail: 478675878@qq.com

Fangteng Du, Department of Gastroenterology, Second Affliated Hospital, Nanchang University, No. 1, Minde Road, Nanchang 330006, China. E-mail: 296978043@qq.com
